# A novel quick transendoscopic enteral tubing in mid-gut: technique and training with video

**DOI:** 10.1186/s12876-018-0766-2

**Published:** 2018-03-13

**Authors:** Chuyan Long, Yan Yu, Bota Cui, Sabreen Abdul Rahman Jagessar, Jie Zhang, Guozhong Ji, Guangming Huang, Faming Zhang

**Affiliations:** 1 Medical Center for Digestive Diseases, the Second Affiliated Hospital of Nanjing Medical University, 121 Jiang Jia Yuan, Nanjing, 210011 China; 2 Key Lab of Holistic Integrative Enterology, Nanjing Medical University, 121 Jiang Jia Yuan, Nanjing, 210011 China; 3 Changshu No.2 People’s Hospital, 68 Hai Yu Nan road, Jiangsu, 215500 China; 4 National Clinical Research Center for Digestive Diseases, Xi’an, China

**Keywords:** Transendoscopic enteral tubing, Mid-gut, Fecal microbiota transplantation, Enteral nutrition, Endoscopy, Nasal-jejunal tube

## Abstract

**Background:**

This study aimed to evaluate the feasibility, safety, and value of a quick technique for transendoscopic enteral tubing (TET) through mid-gut.

**Methods:**

A prospective interventional study was performed in a single center. A TET tube was inserted into mid-gut through the nasal orifice and fixed on the pylorus wall by one tiny titanium endoscopic clip under anesthesia. The feasibility, safety, success rate, and satisfaction with TET placement were evaluated for enteral nutrition or fecal microbiota transplantation.

**Results:**

A total of 86 patients underwent mid-gut TET. The success rate of the TET procedure was 98.8% (85/86). Mean tubing time of the TET procedure was 4.2 ± 1.9 min. 10 cases of procedure was enough for training of general endoscopist to shorten the procedure time (7.0 min vs 4.0 min, *p* < 0.05). 97.7% (84/86) of patients were satisfied with the TET placement. Procedure-related and tube-related adverse events were observed in 8.1% (7/86) and 7.0% (6/86) of patients respectively. There were no moderate to severe adverse events during tube extubation.

**Conclusions:**

TET through mid-gut is a novel, convenient, reliable and safe procedure for mid-gut administration with a high degree of patient satisfaction.

**Trial registration:**

This research was retrospectively registered with clinicaltrials.gov. Trial registration date: 29th November 2017. Trial registration number: NCT03335982.

**Electronic supplementary material:**

The online version of this article (10.1186/s12876-018-0766-2) contains supplementary material, which is available to authorized users.

## Background

In recent years, fecal microbiota transplantation (FMT) has gained appeal as a therapeutic option worldwide. Clinical studies have shown that FMT has a therapeutic role in clostridium difficile infection (CDI), inflammatory bowel diseases (IBD), refractory constipation, chronic diarrhea, liver diseases and metabolic syndrome [1–3].

Traditionally, microbiota can be administered through the upper-gut, the mid-gut, and the lower-gut pathways [4, 5]. FMT via colonoscopy is a classic approach, but in our previous study on ulcerativecolitis [4], those patients have difficulty to maintain the infused microbiota suspension for enough time through this way. Thus, we designed the colonic transendoscopic enteral tubing (TET) technology, which made whole-colon administration of treatment and repeat FMTs possible [5]. However, some patients are resistant to undergo bowel preparation for colonoscopy or some are not suitable for colonic delivering way. Therefore, mid-gut delivering way is an important option for those patients. In our previous researches on FMT for Crohn’s disease [6, 7], patients and physician faced the similar problem that some patients need repeat FMT during hospitalization, and some may need enteral nutrition at the same time.

In order to have a quicker and more convenient placement of mid-gut/nasal-jejunal TET tube than traditional methods, we designed a novel mid-gut TET technique without further confirmation for the location of tube in gut by X-ray or other medical devices after the endoscopic procedure. This study aimed to evaluate the feasibility, safety, and value of the mid-gut TET technique.

## Methods

### Subjects

This prospective interventional study was conducted at the Second Affiliated Hospital of Nanjing Medical University from September 2015 to September 2017. All patients were selected from our clinical trial without payment for the endoscopic devices and met the inclusion criteria, which were age 5–80 years, suitability for endoscopy, and consented to undergo TET placement for their diseases and conditions. Patients were excluded if they had severe diseases due to the risk of anesthesia. Informed consent was obtained from all adult subjects or parents in pediatric cases. The study was approved by the Institutional Review Board of the Second Affiliated Hospital of Nanjing Medical University.

### Preparations, procedures and assessment of TET

**Fig. 1:**
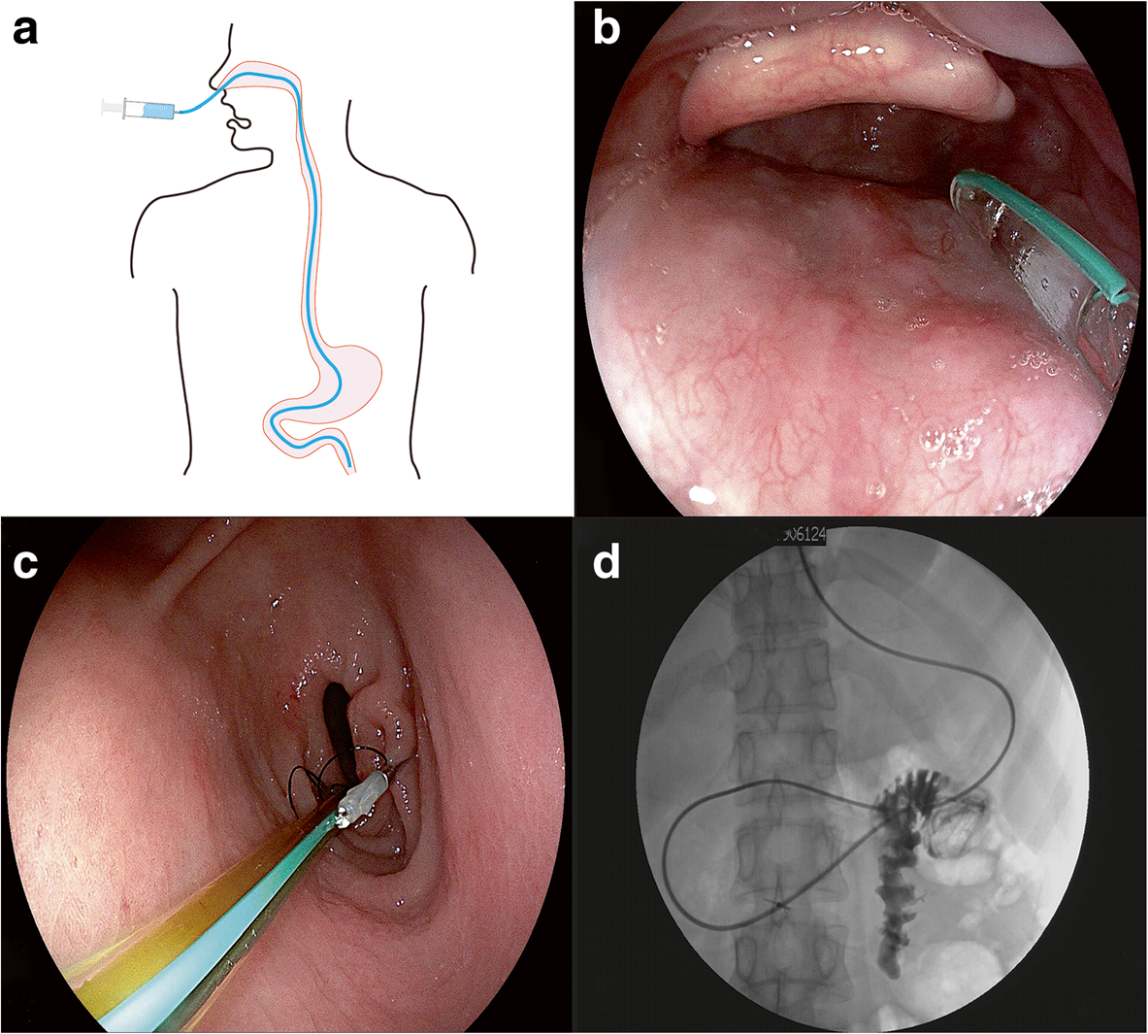
Concept and procedure of administration through mid-gut tubing. **a** The concept of administration through mid-gut tubing; **b** The endoscopic view at mouth cavity when the soft tube tip from nasal cavity was inserted into hypopharynx close to esophagus; **c** One tiny endoscopic clip was used for fixation of the tube at gastric antrum before the guide wire within the tube was removed out; **d** The location of mid-gut tube could be confirmed by X-Ray and contrast agent, but is not necessary using this TET technique and device


Patients underwent the procedure under anesthesia. All patients need this mid-gut tube for frequent fecal microbiota transplantation and/or enteral nutrition support. Two to three mL of liquid paraffin oil (medical use level) was injected into TET tube and then the matched guide wire was inserted into the tube (FMT-DT-N-27/1350, FMT Medical, Nanjing, China). Then the tube was coated with paraffin oil by medical gauze and was inserted into the esophagus through nasal orifice under gastroscopic vision in oral cavity (Fig. 1a, b). The endoscope was then synchronously advanced to the stomach following the tube. The tube should be advanced into the distal duodenum with or without assistant of grasping forceps. As shown in Additional file 1: Video 1, the tube was fixed on the pylorus wall by one titanium clip (Fig. 1c) when the targeting circle (25 cm or 20 cm to the distal tip of the tube) for fixation was located at the pylorus. The endoscopy assistant must hold the tube for avoiding any migration while the endoscope is being slowly withdrawn. After the fixation, the guide-wire should be pulled out partially until the tip of the guide wire within the tube was pulled into the stomach (almost 25–30 cm), which could be confirmed under endoscopic vision. The endoscope should be inserted into duodenum for confirming no buckling changes of the soft tube within the intestinal cavity. The endoscope could be taken out of the body with the stable controlling of the tube from the assistant. The guide wire was required to be taken out of the tube slowly after the endoscope was out of mouth. Finally, the medical tape was used to fix the tube on nose. According to our pilot study, the 100% (total 10 cases) of the distal tube could arrive at the target location (jejunum or distal duodenum) during the development of this device and technique. Therefore, there was no necessary to confirm the location of the distal tip of the tube under fluoroscopy (Fig. 1d). The endoscopic procedure was well-shown from the (Additional file 1: Video 1).

Additional file 1:The endoscopic procedure was well-shown from the video 1. (AVI 18990 kb)


The primary aim of the study was rate of success on the tubing procedure. The secondary aim was rate of adverse events. The end point was 1 month after procedure. The duration of the procedure from nasal intubation to fixation of the titanium clip on the gastric wall was recorded as tubing time. Two endoscopists with different training experience performed the endoscopic procedures in this study. One endoscopist was at advanced level and another one was an endoscopist at general level who had finished about 300 case of endoscopy. In order to evaluate the difficulty of training for TET. The mean time of procedure were compared between and within the two endoscopists. After the fixation, further scanning of the stomach and esophagus or possible biopsy could be performed, but the time was not included. Procedure-related and tube-related adverse events, patient-reported discomfort and satisfaction on TET placement were also recorded. The grade of satisfaction was clarified as yes or no.

### Statistical analysis

All statistical analyses were performed using SPSS software (version 19.0; SPSS Inc., Chicago, IL). Comparison of operating time and adverse event rate between two endoscopists was done with independent sample T-test and Chi square test, respectively. *P* value < 0.05 was considered to be statistically significant.

## Results

As shown in Table 1, total 86 cases underwent transendoscopic enteral tubing in mid-gut. Forty-three males and 43 females aged 5 to 77 years (mean ± SD, 37.1 ± 17.8). Of the 86 patients, the success rate of tube placement was 98.8% (85/86). There was only one failed attempt in one Crohn’s disease patient with gastric-duodenal fistula and the reinsertion rate was 1.2%. The mean procedure time (from beginning of tube inserting into the esophagus to the tube was fixed on the pylorus wall by one titanium clip) was 4.2 ± 1.9 min (range, 1.53–11.25). The mean time of procedure was 3.3 ± 0.9 min and 5.5 ± 2.4 min for the advanced endoscopist and general endoscopist (*p* = 0.002), respectively. The mean time of the initial 10 patients for general endoscopist was longer than the time during the following 10 cases (4.0 ± 1.0 vs 7.0 ± 2.4 min, *p* = 0.015). 20 (23.4%) patients were placed with tubes successfully without assistant from grasping forceps. Procedure-related adverse events included mild pharynx bleeding (1.2%), limited epistaxis (4.6%) and unplanned extubation during post anaesthetic recovery (2.3%). Tube-related adverse events included moderate-severe pharynx discomfort (4.6%), rhinorrhoea (1.2%), and nausea (1.2%). All patients were followed up for 1 month after discharged. Patient-reported satisfactory rate was 96.5% (83/86). Only three (3.4%) patients complained of obvious pharynx discomfort that they do not want to experience again.

**Table 1:** Characteristics of patients undergoing TET through mid-gut

Item	Value
Total number	86
Age, mean ± SD, years	37.1 ± 17.8
Male, n (%)	43 (50.0)
Diseases	
Inflammatory bowel disease, n (%)	36 (41.9)
Others, n (%)	50 (58.1)
TET success rate, n (%)	85 (98.8)
Tubing time, mean ± SD, min	4.2 ± 1.9
Advanced endoscopist, mean ± SD, min	3.3 ± 0.9
General endoscopist, mean ± SD, min	5.5 ± 2.4
Aim of TET, n (%)	
FMT, n (%)	65 (75.6)
Mini-FMT, n (%)	27 (31.4)
Enteral nutrition, n (%)	20 (23.3)
Satisfaction survey for TET, n (%)	83 (96.5)
Adverse events of patients, n (%)	
Procedure-related	
Mild pharynx bleeding, n (%)	1 (1.2)
Epistaxis, n (%)	4 (4.6)
Unplanned extubation, n (%)	2 (2.3)
Tube-related	
Nausea, n (%)	1 (1.2)
Pharynx discomfort, n (%)	4 (4.6)
Rhinorrhoea, n (%)	1 (1.2)

*FMT* fecal microbiota transplantation, *TET* transendoscopic enteral tubing

## Discussion

Traditionally, the nasojejunal tube can be placed blindly, with fluoroscopic or electromagnetic guidance, or more commonly, endoscopically. Blind placement of feeding tube beyond the pylorusis frequently unsuccessful and may lead to complications such as pneumothorax and pneumonia due to misplacement in the bronchus [8, 9]. The success rate was reported 69 to 98% for electromagnetic (EM)-guided placement and 82 to 100% for endoscopic placement [10–16]. In our study the success rate of intubation was 98.8%, superior to the Gerritsen’s research [10] in which the successful placement of nasoenteral tubes was achieved in 3202 of 3789 (85%) with EM-guided, 706 of 793 (89%) with endoscope, and 413 of 446 (93%) with fluoroscopic procedures. Especially for endoscopic placement, the rate of successful placement in this study (98.8%) was much higher than Hirdes and Wildi’s researches (124 of 143 (87%) [17] and 132 of 157 (84%) [18], respectively).

There was only one failed attempt in a patient with Crohn’s disease who had gastric duodenal fistula, and finally, we reinserted the tube with fluoroscopy. The reinsertion rate was 1.2%, which is much lower than reported [10] 21% of EM-guidance (270 of 1279), 16% of endoscopy (64 of 394), and 26% of fluoroscopy (10 of 38).

Additionally, Gerritsen’s [19] reported that only 36% of the patients with EM guidance and 71% of the patients with endoscopy avoided replacement procedure. The initial tube placement was successful in 58% with EM guidance and 53% with endoscopy (*P* = 0.71), much lower than in our research (98.8%). Hirdes’ research [17] consider that the more accurate post-pyloric tube placement can reduce the rate of repeat endoscopies.

The procedure time in our study was 4.2 ± 1.9 min, more time saving than the time from the research [10] on EM-guided placement (13.4 [12.9] minutes), which followed by endoscopy and fluoroscopy (14.9 [8.7] and 16.2 [23.6] minutes, respectively). Although EM-guided placement of nasojejunal feeding tube was used world widely for its convenience of beside placement by nurses [20], this electromagnetic device was not widely used in China. An important thing that should be highlighted: patients can benefit from the endoscopic procedure for possible lesion scanning in upper-gastrointestinal tract. Additionally, the cost of endoscopy and related anesthesia in China is cheap. This could be an important reason for us to have this TET, instead of choosing an expensive tube and special guidewire with electromagnetic detection or more X-ray confirmation.

Procedure-related and tube-related adverse events were recorded in 7 of 86 (8.1%) and 6 of 86 (7.0%) patients in our procedure, respectively. And there was no significant difference on rate of adverse events between two endoscopists (*p* = 0.94). The incidence rate of tube-related adverse events was far less than that with EM-guided, endoscopic, and fluoroscopic placement procedures [10]. Two patients removed the tube unconsciously post anesthesia which might be related to the discomfort of nasal or throat. Thus we suggest that patients should be gloved with restraint mitts during the time of post anaesthetic recovery. There were no tube fracture or leaking, dislocated or blockage in our study. All patients were followed-up within 1 month after discharged, and no more adverse events were observed.

**Fig. 2:**
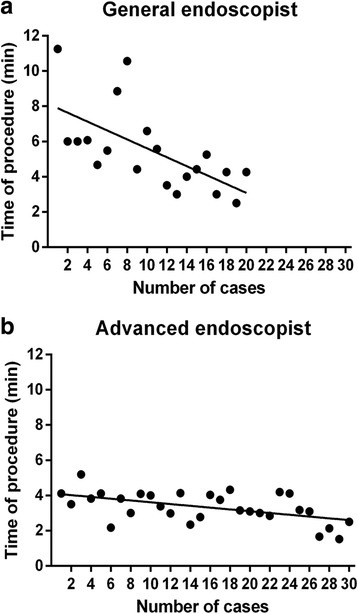
The tendency of operating time in two endoscopists. **a** The time of procedure for the general endoscopist showed significant decreasingtendency during the training; **b** The time of procedure decreased slightly during the training in the advanced endoscopist

Two endoscopists received basic TET skills training before this study. The mean procedure time of 3.3 min for the advanced endoscopist was shorter than 5.5 min for the general endoscopist. As number of cases increased, the time of procedure showed a decreasing tendency (Fig. 2a). This suggest that the experience of endoscopistis an influential factor that can affect the time of operation. This study indicated that 10 cases of procedure might be enough for training of general endoscopist to shorten the procedure time.

Inadvertent removal or displacement of the tube is common in conventional procedures [21, 22], and subsequently additional endoscopy or radiation would be necessary for reposition. In our study, the intubation procedure was carried out under direct vision, and the titanium clip was used to fix the tube and avoid migration.

The present study demonstrated that this TET should be a convenient and economic way to undergo the frequent FMTs and enteral nutrition support. In this study, 65 (75.6%) of patients underwent TET for FMT. 27 (31.4%) patients underwent TET were for mini-FMT (a synthetic formula bacteria with specific bacterial species and stable formula). These data indicated that this mid-gut TET might be safe and useful for providing a new delivering way of microbiota transplantation.

There is no necessary to exchange the tube location from mouth to nasal orifice according to the designed concept of mid-gut TET. Based on our preliminary research during the earlier phase, we confirmed that there was no necessity to confirm the location of the tube by X-ray examination. If the doctor would like to confirm the location of the inserted tube at any time, successful suction of yellow-green bile, pH test for the suction fluid or X-ray could be used to have a defined answer [23]. The tube can be preserved for weeks, and it can be pulled out easily at any time when necessary. The attached line circle could be observed on the tube. Generally, the clip attached on line circle or not was probably related to the maintaining time of the tube.

Beyond the above discussion, we reviewed the reported indications and contraindications, advantages and disadvantages, success rates of different types of nasojejunal feeding tubes and related technology in Table 2. The distribution of indication for mid-gut TET includes: nasal-jejunal administration of medicine or enteral nutrition, mid-gut microbiota transplantation (including FMT or mini-FMT).

**Table 2:** Comparison of different nasojejunal tube insertion methods

	Success rate (%)	Tubing time(min)	Advantages	Disadvantages
TET	98.8	4.2	Endoscopic view, time saving, no need of switch tube from mouth to nasal orifice, no need of confirmation by X-ray	Interventional endoscopy
Blind insertion [24, 25]	17–68	–	Safe and less cost	Time-consuming, low success rate, need confirmation of the location
DSA [10, 26, 27]	84–96	14.9–17.0	Safe and less cost	Time-consuming, X-ray exposure
EM-guided [10, 28, 29]	43–98	6.2–20.0	Operation by trained nurses bedside, no need of fasting	Time consuming, the evidence for altered upper GI anatomy after surgery is scarce
Traditional endoscopic [10, 13, 15, 16, 30]	36–100	6.6–28.0	Under endoscopic view	Interventional endoscopy, time consuming, tube location changing, switching tube frommouth to nasal orifice

*TET* transendoscopic enteral tubing, *DSA* digital subtraction angiography, *EM* electromagnetic

This study had limitations, including not large sample size, not randomized controlled study and no cost-effective analysis. Meanwhile, recordings only from the two endoscopists were collected for analysis. The time of procedure was affected by difficulty of technique, and was also influenced by the individual differences. The comprehension and practical ability of endoscopist cannot be ignored. Further studies are necessary in the future for a more clear conclusion.

## Conclusions

This novel TET technique is convenient, time-saving, and safe way for providing endoscopic mid-gut tubing for frequent microbiota transplantation and nutrition delivering. Moreover, this technique is easy for endoscopists learning and training practice.

## Abbreviations

CDI: Clostridium difficile infection
DSA: Digital subtraction angiography
EM-guided: Electromagnetic guided
FMT: Fecal microbiota transplantation
IBD: Inflammatory bowel diseases
TET: Transendoscopic enteral tubing
